# Intraoperative video recording in otolaryngology for surgical education: evolution and considerations

**DOI:** 10.1186/s40463-023-00620-1

**Published:** 2023-01-19

**Authors:** Hannah L. Brennan, Simon D. Kirby

**Affiliations:** Faculty of Medicine, Memorial University of Newfoundland and Labrador, St. John’s, Newfoundland and Labrador, 98 Pearltown Rd, St. John’s, NL A1G 1P3 Canada

**Keywords:** Intra-operative video recording, Video-based assessment, Otolaryngology, Medical education

## Abstract

**Background:**

Otolaryngology is a surgical speciality well suited for the application of intraoperative video recording as an educational tool considering the number procedures within the speciality that utilize digital technology. Intraoperative recording has been utilized in endoscopic surgeries and in evaluating technique in mastoidectomy, myringotomy and grommet insertion. The impact of intra-operative video recording in otolaryngology education is vast in creating access to surgical videos for preparation outside the operating room to individualized coaching and assessment. The purpose of this project is to highlight the role of intraoperative video recording in otolaryngology training and elucidate the challenges and considerations associated with implementation.

**Methods:**

Related publications between 1999 to 2022 were reviewed from PubMed and Embase databases utilizing search terms “intraoperative videography,” “video recording surgery,” “otolaryngology,” and “surgical education.” 109 articles were screened independently by HB and SK, by title and abstract then full text review. 28 articles from the original search and 6 from the secondary reference review were included.

**Results:**

The application of intraoperative video recording is evident in otolaryngology surgeries including endoscopic sinus surgery, laryngeal surgery, and other endoscopic procedures. There have been significant advancements in recording tools, including devices that can capture the surgeon’s perspective. The considerations and challenges identified with utilizing this educational tool were categorized into different themes including ethics/consent, regulation, liability, data, technology, and human resources.

**Conclusion:**

Intra-operative video recording has been demonstrated to have significant impact within otolaryngology education. It is critical to elucidate the challenges and considerations involved to utilize this educational tool effectively. Future directives will see video-based performance analytics providing comparative metrics to encourage precise coaching of surgical residents.

## Background

Intraoperative video recording is frequently used for education, research and quality improvement across various surgical specialties, including general surgery, ophthalmology and orthopedic surgery [1–3]. This tool has been used in minimally invasive surgeries, using laparoscopic or endoscopic recording devices, as well as open surgeries, most commonly within general surgery [4]. The wider adoption of intraoperative video recording, particularly within general surgery, provides insight to inform the further implementation with Otolaryngology.

Otolaryngology is a surgical speciality well suited for the application of intraoperative video recording as an educational tool considering the number procedures within the speciality that utilize digital technology with recording capability. Surgical videos have also been used in surgical preparation and mastery of surgical anatomy in many otolaryngology procedures including otological surgeries, laryngoscopy, thyroidectomy, neck dissection, rhinoplasty and rhytidectomy. [5–10] Intraoperative recording has been utilized in assessment of technical skills in various surgeries including, myringotomy and grommet insertion, mastoidectomy and endoscopic sinus surgery [11–13]. The impact of intra-operative video recording in otolaryngology education is vast in creating access to surgical videos for preparation outside the operating room for individualized coaching and assessment. The purpose of this project is to highlight the role of intraoperative video recording in otolaryngology training and elucidate the challenges and considerations associated with implementation.

## Methodology

**Fig. 1:**
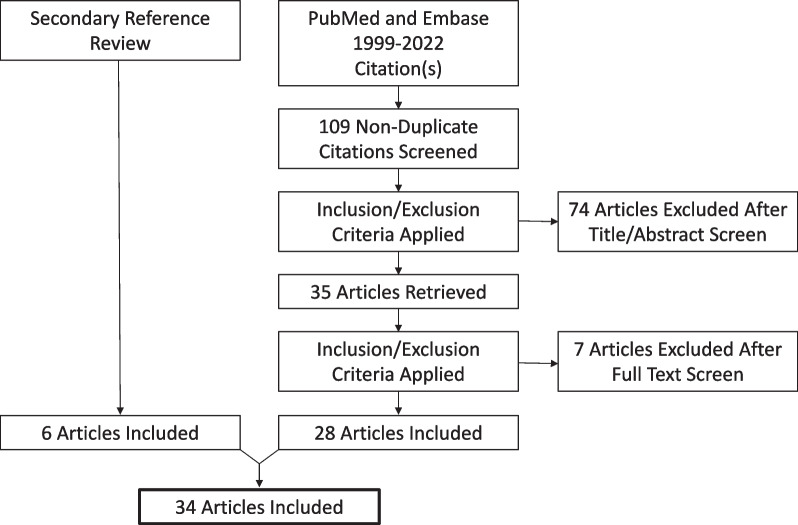
PRISMA Diagram summarizing search strategy

Related publications between 1999 to 2022 were reviewed from PubMed and Embase databases utilizing search terms “intraoperative videography,” “video recording surgery,” “otolaryngology,” and “surgical education.” The search strategy is outlined in Fig. 1. Both authors, H.B and S.K, completed the screening process. 109 articles were screened by title and abstract then full text review, resulting in including 28 articles from the original search with 6 articles added from secondary reference review. The inclusion criteria involved is outlined in the PICO framework below and involves studies focused on intraoperative video recording for resident education within otolaryngology surgeries. Non-English articles were excluded from this review.

(P) The population focus of this study was residents in otolaryngology head and neck surgery. (I) The intervention analyzed was intraoperative video recording, for education and assessment. (C) The use of intraoperative video recording was compared to traditional educational resources and assessment approaches. (O) The major considerations and challenges of implementing intraoperative video in otolaryngology education and assessment were identified in terms of technology, data, human resources, ethics/consent, regulation and liability.

## Main body

### Intraoperative video recording in otolaryngology education: surgical preparation and knowledge

Operative videos have been increasingly employed within otolaryngology training. The need for this resource has been highlighted by the COVID-19 pandemic which impacted hands-on training. Access to surgical videos is an important resource for medical students, residents, and practicing surgeons seeking continuing educational opportunities, as well as surgeons in countries with various levels of training resources. In a survey of resident trainees, the resource of surgical video library was utilized most frequently in the preparation for upcoming cases, but was also used for general surgical anatomy learning and solidifying concepts after being involved in a case [14]. A small pilot study displayed the utility of otological surgical videos in resident education, particularly as a resource for preparation for surgery [5].

The educational value of utilizing surgical video has also been elucidated in a number of studies. One study demonstrated the value of intraoperative video in learning surgically relevant head and neck anatomy with two groups of medical students utilizing novel surgical video atlas for thyroidectomy compared with traditional textbook resources. The video atlas arm scored higher in the post test and were more satisfied with their learning compared to those using traditional textbook resources [15]. A study displayed that diagnostic interpretation of flexible transnasal laryngoscopy can improve with video teaching of laryngoscopies, particularly for assessing vocal cord mobility [6].

Intraoperative video recording has been utilized in the development of a teaching module for thyroidectomy surgery and neck dissection, utilizing a 3-camera viewing system for multiple vantage points. These modules were assessed using intraoperative video as well, in which residents wore a headlight camera while performing a thyroid lobectomy and neck dissection before and after watching the teaching module [7, 8]. The de-identified residents’ footage was analyzed and displayed a decreased in procedural errors following the thyroidectomy module. Procedural errors reflect inaccuracies executing the predetermined component steps in the correct order. Interestingly the executional errors or faults in manipulation of surgical instruments, did not significantly decrease with the module, indicating a further role for coaching and review of intraoperative video [7]. The neck dissection module was also assessed using de-identified intraoperative video and scored using the Observational Clinical Human Reliability Assessment (OCHRA) system and reported a reduction in procedural and execution errors, with a total of 55% decrease in error occurrence. [8]

### Intraoperative video recording in otolaryngology education: assessment

Otolaryngology residency programs utilize various assessment tools, including case logs, oral and written examinations as well as evaluations under supervision in the operating room. Recently surgical education has shifted towards competency-based medical education (CBME), which emphasizes assessment to track progress [16]. A study conducted with faculty and residents in otolaryngology residency programs demonstrated a need for innovation and increased structure in the approach to peri-operative teaching and feedback. Intraoperative video recording provides an opportunity to address this issue providing an avenue for structured feedback, objective comprehensive assessment and monitoring development of surgical technical skills [17].

One of the strengths of utilizing intraoperative video in assessment and evaluation is how multiple independent evaluators can assess the resident’s surgical skills, creating a reliable and valid evaluation system. Another benefit of this assessment approach is allowing for objective, unbiased evaluation of surgical skills by blinding the identity of the resident to the evaluator. Additionally, many surgeries with small operative fields and microscopic endoscopic approaches can be difficult for supervisor to visualize while assessing trainees.

A study by Bowles et al. evaluated the use of intra-operative video recording as an objective assessment tool for myringotomy and grommet insertion [11]. The study reported strong inter-rater correlation, indicating high reliability of the video assessment. The time to complete the procedure was also measured and found a significant inverse relationship between the time taken to complete the procedure and the mean score allocated [11]. Another study also employed intraoperative video recording in the identification of human error in myringotomy and ventilation tube (VT) insertion. Identifying the common errors, including failure to perform a unidirectional myringotomy incision and multiple attempts to place VT, can aid in training review and educational feedback [18]. Another study demonstrated how assessment tools, like a task-specific checklist (TSC) and global rating scale (GRS), can be used with intraoperative video recording to standardize assessment of myringotomy and tympanostomy tube insertion performance, with inter-rater and intra-rater reliability above 0.88 [19].

A study evaluating surgical technique in mastoidectomy, which blindly evaluated intra-operative video for junior and senior residents and attending surgeons was also reviewed. The study demonstrated reasonable metrics for evaluation of surgeon skill level including drill stroke count, drilling efficiency, stroke pattern and use of suction irrigator. As evaluation and assessment can be time consuming, this study also highlighted how short video segments can provide valuable information on skill level [20]. Another study also focused on intraoperative recording of mastoidectomies for assessment, found that using objective metrics were more accurate than subjective assessment in differentiating surgeon experience level. Additionally the study used software to track the drill, suction irrigator and patient head to provide objective metrics, and found significant differences between faculty surgeons and residents [12].

A video-based assessment tool was developed at John Hopkins Hospital for evaluation of surgical skills in endoscopic sinus surgery (ESS), utilizing intraoperative video recording and assessment checklists [13]. There were significant differences in performance noted between junior and senior residents. This was demonstrated to be a time effective assessment model taking on average 20 min for evaluators to assess the video, which was notably significantly less time than would be required in an in-person evaluation [13]. Another benefit of video-based surgical assessment includes a potential increase in patient safety and operating room (OR) efficiency, as the attending physician can focus on patient care and review the trainee’s performance thoroughly outside the OR [21].

This form of assessment also allows for incorporation of artificial intelligence (AI) to provide a deeper understanding of the surgical skills with less required human analysis. Intraoperative tool movement tracking data has been shown to be clinically useful in quantifying surgical performance. A study demonstrated that machine learning can be utilized to identify surgical instruments within endoscopic endonasal intraoperative video and increase access to this information of surgical performance [22]. Deep neural networks have also been used in analyzing the operative steps in laparoscopic sleeve gastrectomy, with a 85.6% accuracy validated against surgeon annotations of the videos [23]. The application of computer vision, a form of AI, also has the potential to innovate surgical assessment with intraoperative footage.

### Intraoperative video recording in otolaryngology education: coaching

Along with assessment, intraoperative video recording also allows for coaching. This approach has been implemented in sports settings, where coaches review plays with team members following a game to identify areas of improvement [24]. This coaching tool has been widely studied in other surgical specialties, particularly general surgery. A study with general surgery residents demonstrated how this coaching tool can supplement intraoperative learning, providing more individualized instructions and increasing the depth of the teaching points, particularly in regards to surgical decision making compared in intraoperative teaching alone. [25]

As patient safety and outcomes are directly related to surgical performance and technical skill, video-based coaching (VBC) has the potential to identify individual areas of improvement and subsequently impact quality of care and safety. VBC has been demonstrated to impact skill acquisition, as surgical residents who received VBC had significant improvements compared to the residents who did not receive this video coaching when evaluated with standardized assessments [26]. Another study in laparoscopic surgical trainees demonstrated the utility of VBC as participates who received VBC significantly outperformed controls on all global rating scales. [27]

Within Otolaryngology, a recent study evaluated video-based coaching for mastoidectomy education and highlighted the resident-perceived benefit of richer teaching and promotion of a deeper surgical understanding. Notably video-based coaching can be implemented easily in otolaryngology subspecialty surgeries utilizing video-recording capable equipment. [28]

**Fig. 2:**
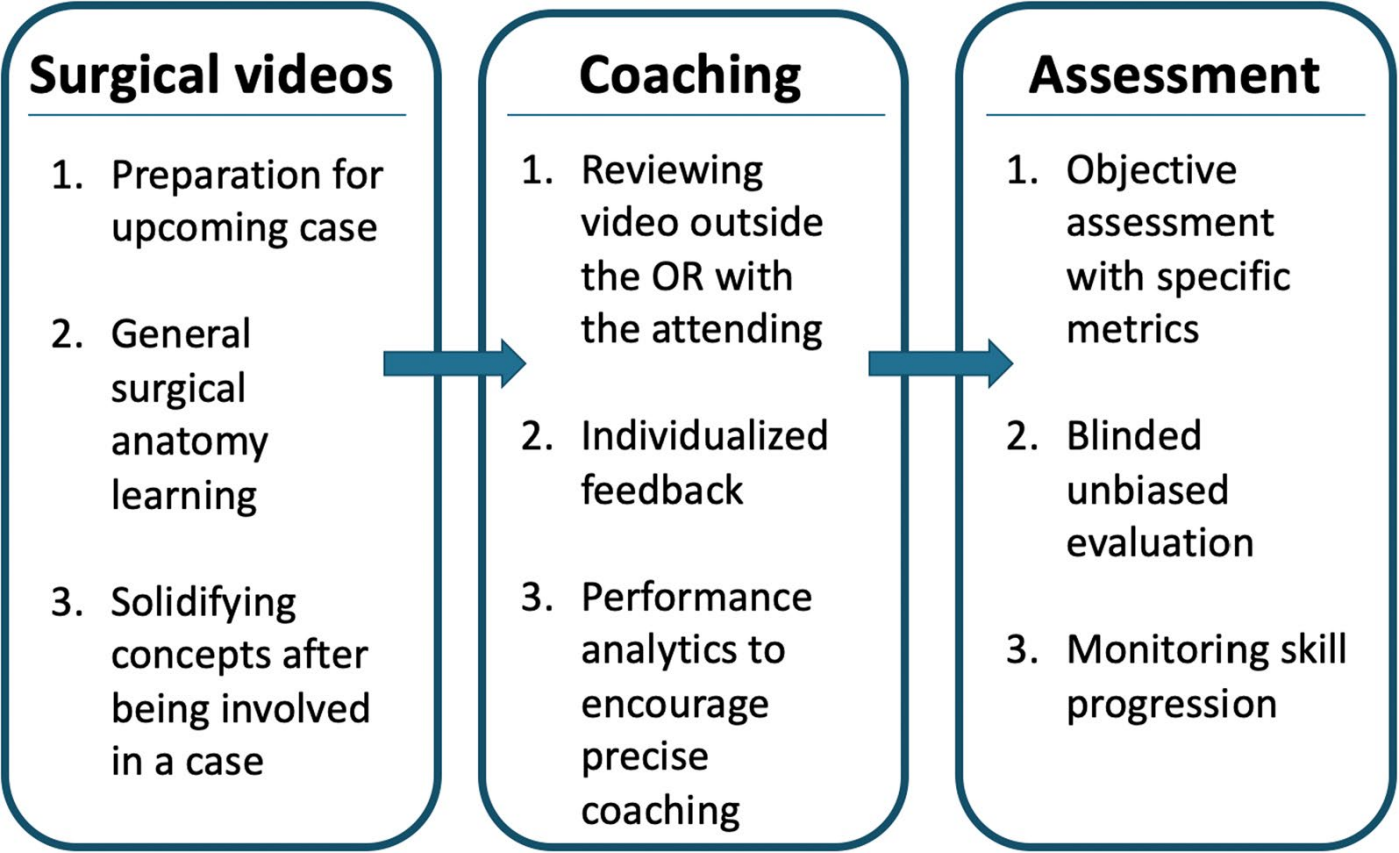
The role of intraoperative video recording in otolaryngology education

The role of intraoperative education is summarized in Fig. 2, highlighting surgical videos, coaching and assessment.

### Considerations and challenges

**Fig. 3:**
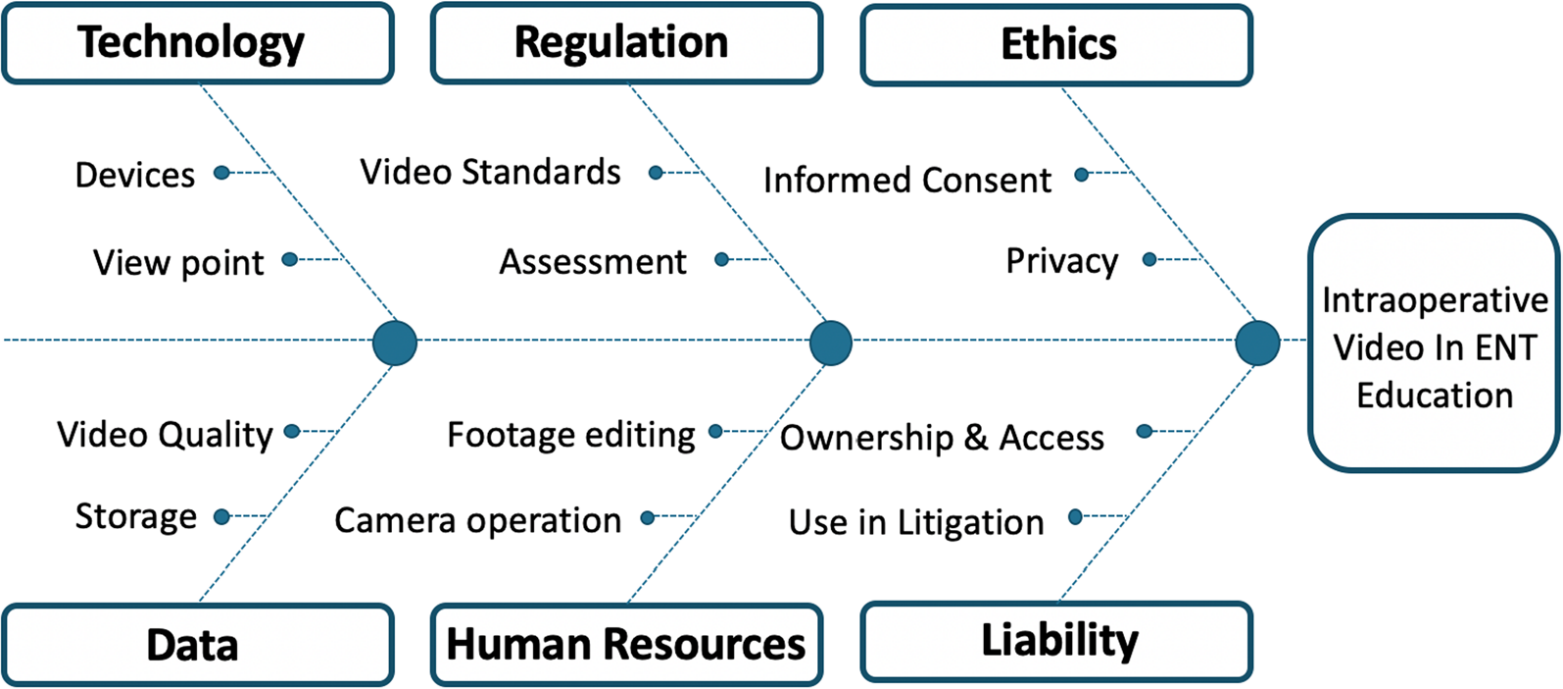
Considerations for implementing intraoperative video recording in otolaryngology education

The considerations and challenges associated with implementing intraoperative video recording in Otolaryngology education are outlined below and shown in Fig. 3.

#### Technology

There have been significant advancements in the devices available for recording intraoperatively as well as in the recording techniques utilized. An important consideration in intraoperative recording is consideration of the type of device that will be used and the advantages and disadvantages involved.

The ability to capture the operator’s perspective has had an impact on the educational value of the footage as well. This is especially important in microscopic endoscopic surgical approaches which can be difficult for supervising surgeon to visualize. GoPro head mounted cameras have been utilized on the surgeon and surgical assistant to provide different optimal views of otolaryngology surgery and gather footage which can aid in the education of both surgeons and surgical assistants [29]. Another study compared the use of two other intraoperative video devices in head and neck reconstructive surgery [30]. The Osmo Pocket was found to be a cost-effective tool to provide first person perspective of the surgery and continuous vision of the operative field, and was limited by the operator’s comfort with the head mounted position and a lack of zoom system [30]. The Vitom device allowed for higher quality images at a higher cost and required repositioning of the camera throughout the surgery [30]. Another study demonstrated the ability of a prototype video device to provide the exact perspective of the microsurgeon and magnify the view through the loupe in thyroid surgery [31]. Google Glass is another device that captures the perspective of the wearer. It can play a role in surgical education and allow trainees to visualize the small surgical field by streaming the video to a computer screen in real time. [32]

The use of mobile smartphone devices has also been evaluated as a method for intraoperative recording of various head and neck surgeries including submandibular gland resections, neck dissections, and supraglottic laryngectomy [33]. An iPhone with an app designed for recording open surgical procedures, has been used for recording the surgeries with an acceptable image quality. The main limitations to this approach include storage and adjusting the recording to have the surgical field centered throughout the videography [33]. Both mobile smartphone devices and Google Glass are device options that also require further ethical and privacy considerations.

#### Ethics and consent

In order to implement intraoperative video recording into otolaryngology training ethics, consent and privacy of the patient need to be considered.

It is also necessary to review the impact of video recording on OR staff. Staff attitude regarding implementing recording initiatives has been assessed and categorized into themes of safety culture, imposter syndrome and privacy concerns [34]. Another study demonstrated that these concerns are successfully addressed with post-processing and de-identifying the footage. [35] It is evident that it is possible to maintain anonymity of staff while still producing video retaining the surgical activity details for educational use.

Informed consent of the patient is vital in the ability to perform a surgical procedure. Before video recording takes place within the operating room, the patient must understand the purpose for recording, the intended audience, where and how long the data will be kept and the procedures in place to de-identify the information and protect privacy [36]. The question of who has ownership of the data also needs to be addressed to discuss consent as well. The ability to withdraw consent at any time should also be communicated to the patient and an approach to ensure deletion of the captured footage must also be in place [37].

As previously mentioned, some of the devices that can be used for videography have communication features that are critical to be cognizant of to implement the necessary precautions to protect patient privacy. The study that utilized a mobile smart phone demonstrated this by using a phone without a sim card and Wi-Fi connection and storing the data in a secure fashion without identifying information [33]. Notably the Google Glass devices makes ensuring patient privacy more difficult as it has the ability to access the internet and communicate with others using voice commands, creating potential for breaches in privacy-protected health information. Obtaining the patient’s informed consent and ensuring privacy throughout the video recording, including proper draping of the patient and avoiding any identifying patient information would be critical before using this device [32]. The evolution in devices also underlines the balance between technological advancement and maintaining privacy. Additionally, if audio recording is also a component of the data collection, there is a risk of confidentiality breeches through conversations between the surgeon, patient, and OR staff. This can be mitigated by avoiding audio recording and adding verbal commentary post-operatively if required [37]. A survey of gynecologists, urologists and residents reported 63.8% of respondents preferred the use of only video recording, without audio, when this tool is implemented in the OR. [38]

It is also critical to consider the resident and surgeon perspective in terms of ethics, consent and privacy with intraoperative video use. In the same way, informed consent is required from the patient it is also essential to obtain from the resident and surgeon involved as well. Defining the use of the video data for educational purpose, who will have access, where and how long it will be stored also need to be address in this context. Answering these important questions prior to using this tool can ensure that can concerns about how the data may be used in residency assessment and impact future career endeavors, potentially serving as a means to demonstrate skill competency.

Video Recording in the OR can be used for education, research, and quality improvement and clarifying the purpose of the data is essential in regard to ethics, consent and medicolegal concerns. Surgical video recording has also been shown to be associated with reduction in errors and positive impact on patient safety [39]. The privacy and medicolegal concerns, discussed further in the next section, have to be balanced against the potential benefit that can be offered from intraoperative video recording, in terms of resident education and subsequent improved patient outcomes.

#### Regulation and liability

Regulation is important to consider in the educational application of intraoperative video considering the videos that are available, the methods and approach to videography and the implementation in assessment.

With multiple devices available, the importance of standardizing the video method for optimal view and positioning for educational purposes is evident. Optimal videography also varies between procedures and the unique challenges associated with different subspecialty surgeries. Rhinoplasty, for example, has challenges including a small surgical field with limited sightlines and requires unique viewing angles which vary throughout the procedure. An analysis of the video standards for rhinoplasty education identified that upward camera angle has been most frequently used and endoscopic view was least frequent [40]. Another study has also been conducted to review the most effective videography position to visualize the nasal-dorsal part of rhinoplasty [9]. Within general surgery, guidelines have been developed for reporting of educational videos in laparoscopic surgery, in regards to video quality, case presentation, demonstration of the surgical procedure, to provide regulation and standardization of this resource [41]. Similarly, there is a need in otolaryngology for guidelines to indicate the best view angles for different aspects of the procedure under consideration to optimize and standardize the recordings for educational use.

As Intraoperative video recording can allow for objective assessment of residents, there is a need for regulation of the assessment approach to promote standardization. The question of whether to edit the footage that is being evaluated is important to consider [16]. The advantage of editing is a shorter assessment time and only assessing the critical technical sections of the surgery. Another option is not editing the video but allowing the evaluator to fast-forward, allowing for faster assessment again. Both would also require further standardization of what these critical sections are in the various surgeries and what sections can be fast-forwarded. Additionally, editing or fast-forwarding also limits the ability of the evaluator to gauge the flow of the operation and assess communication skills that are clear in live assessment [13]. The implementation of artificial intelligence into the assessment and analysis of intraoperative video provides an avenue to access greater metrics or information regarding the skill assessment in a timely manner.

It is also important to consider liability and potential legal impact of intraoperative video recording. There is potential for the recording to be utilized in defense or prosecution of the surgeon when implicated in negligence or misconduct. There are laws that protect information collected for quality improvement from being used as evidence in malpractice lawsuits through nondisclosure and confidentiality rights [36]. Clearly answering and exploring the questions of who has access to the data, and what can the data be used for are critical and key considerations.

Outlining how the video data can be used, in this case for education of surgical trainees, is critical in terms of the medicolegal perspective. If the intent for the data collected was for quality improvement or research purposes, the ownership of the information would be the hospital and physicians for case review, clinical research and protected from litigation [42]. If the data were to be used as a part of the patient’s medical record, then it would be available for litigation in malpractice cases. If an error did occur in the OR, this data may offer an opportunity to learn from and address unanticipated mistakes and potentially create early resolution or alternative pathway than formal litigation [42]. While medicolegal concerns have led to some hospitals ceasing the use of intraoperative video recording, there have also been cases that demonstrate video recordings to lend legal support to the healthcare worker or surgeon [39]. In one case video recording was used to provide supportive evidence of the standard of care and value of patient safety, with documentation of all surgical steps performed accurately and appropriate counts and procedures followed to ensure all equipment was accounted for. [43]

#### Data

One of the major considerations in intraoperative video as an educational tool is the quality of existing data and videos that are publicly available. Publicly available surgical videos on YouTube have been assessed in a number of studies. A study reviewed the available surgical videos for rhytidectomy on YouTube and found variability in the videos, many lacking discussions of key aspects of successful surgery and concluded that discretion should be utilized when accessing such videos as a learning tool [10]. A similar study also echoed the finding that the videos available online for rhytidectomy vary greatly and lacking safeguards to report quality or accuracy [44]. A study also assessed the surgical videos of neck dissections on YouTube and noted the quality of the videos widely varied and reported no correlation between the quality and the age and popularity of the video [45]. Surgical videos in rhinology that were assessable online were found to demonstrate the same heterogeneity in quality and reliability, again highlighting the necessity of standardization and quality review. [46]

Given this variability of intraoperative videos, international recommendations have been created to assist in the creation and standardization of educational surgical videos in otolaryngology and head and neck surgery. These recommendations include ethics considerations anonymizing the patient, technical aspects of editing and high quality, narration, and surgery steps [47]. Another evaluation tool, the Université de Montréal Objective and Structured Checklist for Assessment of Audiovisual Recording of Surgeries/techniques (UM-OSCAARS) was developed to assess the quality of surgical videos for educational purposes. This tool was evaluated with multiple otolaryngology surgery videos and found to have agreement among evaluators, excellent interrater reliability and test–retest correlation. [48]

An open-access comprehensive otolaryngology head and neck surgery video atlas has recently been developed to allow for access to high quality videos narrated with important commentary including surgical steps and key landmarks. [14] Consolidating high quality videos to this atlas with categorization by subspecialty was a major improvement in the accessibility of this educational resource.

#### Human resources

The human resources required to utilize intraoperative video recording is also critical to review. While some devices capture the perspective of the operator with little to no adjustment needed, other devices require angle changes and adjustments throughout the surgery. The personnel required to make such adjustments, set up the devices and maintain the devices would be an additional cost. Additionally, implementing this tool in surgical education also requires personnel to edit the footage as well. Another considerations for video-based assessment includes the time and human resources outside the OR required by attending physicians to review the video and provide individualized feedback [21]. Although this approach has been demonstrated to be time effective with the ability to fast forward and focus on specific aspects of the surgical video, it may be difficult for the attending surgeon to provide this high-quality assessment in a timely manner adding to their responsibilities outside OR time. Despite the evidence of effectiveness of video based coaching, less than 5% of surgical residency programs employ intraoperative video in the operating room [26]. The human resources and time required to develop coaching curricula and integrate video into the operating room is a significant barrier to consider. A survey of American surgical residency program directors demonstrated that programs without video coaching underestimate the utility of this tool in training surgical residents [49]. This demonstrates the need for human resources in respect to education on the utility and effectiveness of this tool, in order to change the current paradigm and aid implementation of intraoperative video in residency training.

## Conclusion

Intra-operative video recording has been demonstrated to have significant impact within otolaryngology education, in terms of its application in knowledge pre-operative preparation, assessment and coaching. It is critical to elucidate the challenges and considerations involved to utilize this educational tool effectively. Future directives will see video-based performance analytics providing comparative metrics to encourage precise coaching of surgical residents.

## Data Availability

Data sharing is not applicable to this article as no datasets were generated or analysed. All material included in the review was accessed through PubMed and outlined in the references section.

## Abbreviations

OCHRA: Observational clinical human reliability assessment (OCHRA)
VT: Ventilation tube
TSC: Task-specific checklist
GRS: Global rating scale
ESS: Endoscopic sinus surgery
OR: Operating room
AI: Artificial intelligence
VBC: Video-based coaching
UM-OSCAARS: Université de montréal objective and structured checklist for assessment of audiovisual recording of surgeries/techniques
